# Aftermath of the Parkland Shooting: A Case Report of Post-traumatic Stress Disorder in an Adolescent Survivor

**DOI:** 10.7759/cureus.6146

**Published:** 2019-11-13

**Authors:** Katy McLaughlin, Jeena A Kar

**Affiliations:** 1 Psychiatry, HCA East University Hospital/Nova Southeastern University, Tamarac, USA; 2 Medical Education and Simulation, Nova Southeastern University School of Osteopathic Medicine, Fort Lauderdale, USA

**Keywords:** ptsd, child and adolescents, news media, social media, parkland, gun violence, ptsd

## Abstract

The Journal of Child and Family Studies states that there have been more mass shootings within the last 18 years than in the entire 20th century combined, with 77% carried out by adolescents. This case study aims to evaluate the clinical presentation of post-traumatic stress disorder (PTSD) in an adolescent by highlighting the clinical course of a school shooting survivor. Here, we present the case of a 15-year-old female who presented to the emergency department (ED) of the University Hospital Medical Center (UHMC) under the Baker Act by the police for self-injury and self-harm. She had been admitted three times to the psychiatric hospital, all following the trauma of surviving the shooting. She met the criteria for PTSD and was triggered by graphics in the news outlets and social media.

As clinicians working with PTSD adolescents, we must be cognizant of these factors as we think about prognosis and create a comprehensive treatment plan. This case study brings to our attention how complex and multifaceted PTSD patients can be in this day and age where social media, news outlets, and television are so pervasive. Three clinical pearls to take home from this patient encounter are: understanding the importance of news and media in modern-day PTSD diagnosis, how certain avoidance behaviors can delay remission, and how uncontrolled re-exposure can lead to poorer outcomes in children and adolescents specifically. It is no longer a rare occurrence to have a patient who has survived a mass school shooting. As a result of this unfortunate reality, clinicians need to be able to recognize and treat symptoms of PTSD in adolescent patients. We must be equipped to expect the interplay of modern triggers such as social media and news media and how it may affect adolescent patients.

## Introduction

According to the Centers for Disease Control and Prevention, there were 39,773 deaths by gun violence in 2017 with only 486 classified as unintentional [1]. Furthermore, the Journal of Child and Family Studies states that there have been more mass shootings within the last 18 years than in the entire 20th century with 77% carried out by adolescents [2]. This care report aims to evaluate the clinical presentation of post-traumatic stress disorder (PTSD) in adolescents. Research suggests that mass shooting survivors may be at greater risk for mental health difficulties compared with people who experience other types of trauma [3-4]. The National Center for PTSD estimates that 28% of people who have witnessed a mass shooting develop PTSD and 1/3 develop acute stress disorder [5].

Furthermore, this report illustrates an important correlation between media coverage on increasing incidents of shootings as well as other triggers found within various types of media and negative effects on long term prognosis among victims with PTSD. In the discussion, emphasis is placed on awareness and prevention of such violence within communities. The cornerstone for change must begin at the front lines - mental health professionals, parents, and importantly survivors within communities. In order to foster a necessary change, there must be a change in how future perpetrators within adolescent populations are identified through proper screening and interventions through resources within the community.

## Case presentation

The patient is a 15-year-old female who presented to the emergency department (ED) of the University Hospital Medical Center (UHMC) (Tamarac, FL) under the Baker Act by the police for suicidal ideation and self-harm. This is the patient’s third hospitalization within the past three months with a similar presentation. Upon admission, she reported excessive worry and anxiety associated with intrusive thoughts related to the shooting at her high school approximately nine months prior. She reported frequent nightmares of the shooter entering a building and killing multiple people. She also reported that these nightmares are now causing her to avoid familiar places (such as her school or her father's workplace) as well as other public places as they induce flashbacks and panic attacks; she has now been having thoughts and urges to self harm by cutting as well as frequent suicidal ideations due to the severity of these symptoms.

She was admitted to the inpatient psychiatric unit where she was seen and assessed daily by the psychiatry team. She received individual therapy by a mental health counselor while on the unit. The therapy was centered around building coping skills. Her parents were counseled on her symptoms and family sessions were conducted by the psychiatry team to educate both the patient and her parents on diagnosis and treatment options. During family sessions, the parents expressed that they were against using medications in children. Techniques such as motivational interviewing (MI) were utilized by multiple providers such as the attending physician and resident. Her parents declined pharmacologic treatment despite MI interventions and she was discharged home with follow up in a youth Intensive Outpatient Program (IOP) the following week post discharge. Youth IOP consists of therapy sessions three days a week, addressing non-pharmacological ways to address her PTSD.

## Discussion

Case-based clinical discussions: three important takeaway points

*1)* *Media Exposure and the Effects on PTSD Related to Mass Shootings*

A study conducted by the National Institute of Mental Health found that media exposure interacted with sympathetic reactivity to predict PTSD symptom onset, such that adolescents with lower levels of sympathetic reactivity developed PTSD symptoms only following high exposure to media coverage of the attack. Although this particular study is in regards to the Boston Marathon bombing, it illustrates the point that media exposure may be particularly likely to trigger PTSD symptoms in youths with exposure to violence [6].

Additional studies in the context of 9/11 terrorist attack support the notion that media exposure can trigger extreme PTSD symptomology. The study conducted by University of Toronto found that exposure to graphic media images may result in physical and psychological effects previously assumed to require direct trauma exposure [7].

A study by the American Academy of Child and Adolescent Psychiatry (AACAP) followed 66 children after a school shooting and found that the media played a role in perpetuating high levels of emotional tension in children. They found that consistent exposure to graphic content led to the formation of “malignant memories" [8].

*2)* *Avoidance Behavior *

Data collected from survivors of the Virginia Tech shooting was collected using the Acceptance and Action Questionnaire-II (AAQ-II), a 7-item self-report measure of experiential avoidance. Avoidance behaviors are any action or behavior taken to prevent difficult or painful feelings. The study found that avoidance behaviors are implicated in the etiology and prolongment of post-traumatic stress symptomatology [9].

*3)* *Delay of Disease Remission*

It may be more difficult to overcome the symptoms of illness (PTSD) due to more frequent and intense triggers within the environment causing further delay of disease remission. According to the Journal of Urban Health, only about one-half of the PTSD cases identified at any time over three years were in remission at the three-year follow-up [10].

**Figure 1:**
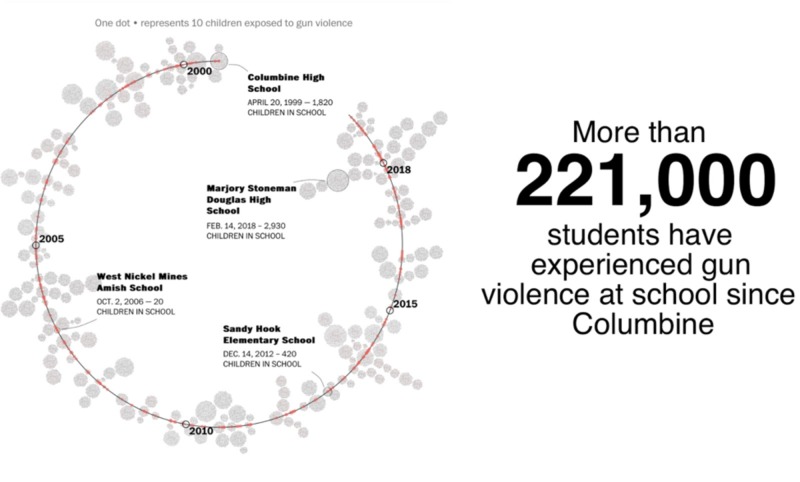
Washington Post Infographic This figure is a graphic representation/schematic by the Washington Post, designed to illustrate the occurrence of shootings and their respective scale on a timeline [11].

As clinicians working with PTSD adolescents, we must be cognizant of these factors as we think about prognosis and create a comprehensive treatment plan. In the figure below, we see that gun violence has been increasing, and we must be equipped to deal with the long-term sequelae of these events, given how unfortunately common they have become (Figure 1) [11].

Given the limited amount of research conducted on gun violence [12], it is paramount for cases such as these to be documented and added to medical literature so that the rippling effects of mass shootings can be understood on a clinical level. This case study brings to our attention how complex and multifaceted treating mass shooting survivors can be. PTSD patients are exposed to their trauma differently in this day and age where social media, news outlets, and television are so pervasive. As news outlets sensationalize the reported tragedies, we must focus our attention on our patients and providing the best care and support in dealing with these factors.

## Conclusions

This case study and discussion show that it is no longer a rare occurrence to have a patient who has survived a mass school shooting. As a result of this unfortunate reality, clinicians need to be able to recognize and treat symptoms of PTSD in adolescent patients. In addition to appropriate medications and psychotherapy, the clinician needs to be prepared to face barriers to remission such as re-exposure through media content. This case study also prompts physicians to recognize avoidance behaviors of such social media and news content as a manifestation of PTSD in adolescents. This case serves as an example of the long term sequelae of PTSD, and could be the springboard for further advocacy and awareness about the aftermath of school shootings.

## References

[1] (2019). Underlying cause of death 1999-2017. https://wonder.cdc.gov/wonder/help/ucd.html.

[2] Katsiyannis A, Whitford DK, Ennis RP (2018). Historical examination of united states intentional mass school shootings in the 20th and 21st centuries: implications for students, schools, and society. J Child Fam Stud.

[3] Kann L, McManus T, Harris WA (2018). Youth risk behavior surveillance — United States, 2017. MMWR Surveill Summ.

[4] Miron LR, Orcutt HK, Kumpula MJ (2014). Differential predictors of transient stress versus posttraumatic stress disorder: evaluating risk following targeted mass violence. Behav Ther.

[5] Novotney A (2018). What happens to the survivors: long-term outcomes for survivors of mass shootings are improved with the help of community connections and continuing access to mental health support. APA.

[6] Busso DS, McLaughlin KA, Sheridan MA (2014). Media exposure and sympathetic nervous system reactivity predict PTSD symptoms after the Boston Marathon bombings. Depress Anxiety.

[7] Silver RC, Holman E, Andersen JP, Poulin M, McIntosh DN, Gil-Rivas V (2013). Mental-and physical-health effects of acute exposure to media images of the September 11, 2001, attacks and the Iraq War. Psychol Sci.

[8] Schwarz ED, Kowalski JM (1991). Malignant memories: PTSD in children and adults after a school shooting. J Am Acad Child Adolesc Psychiatry.

[9] Kumpula MJ, Orcutt HK, Bardeen JR, Varkovitzky RL (2011). Peritraumatic dissociation and experiential avoidance as prospective predictors of posttraumatic stress symptoms. J Abnorm Psychol.

[10] North CS, McCutcheon V, Spitznagel EL, Smith EM (2002). Three-year follow-up of survivors of a mass shooting episode. J Urban Health.

[11] Cox Cox, J. (18, April 20 (2019). Explore the Washington Post's database of school shootings. https://www.washingtonpost.com/graphics/2018/local/school-shootings-database/?noredirect=on&utm_term=.d1b8c6eacdee.

[12] Kellermann AL, Rivara FP (2013). Silencing the science on gun research. JAMA.

